# ORM1 promotes tumor progression of kidney renal clear cell carcinoma (KIRC) through CALR-mediated apoptosis

**DOI:** 10.1038/s41598-023-42962-w

**Published:** 2023-09-21

**Authors:** Gang Yu, Juan Gao, Weifeng Hu, Dayong Hu, Weibing Wang, Shiquan Yang, Jing Gao

**Affiliations:** 1 Department of Nephrology, The Sixth People’s Hospital Affiliated to Shanghai Jiaotong University, Shanghai, 200233 China; 2 Physical Examination Center, Shanghai Lung Hospital Affiliated to Tongji University, Shanghai, 200433 China; 3 Department of Nephrology, PLA Naval Medical Center, Shanghai, 200052 China; 4 Department of Nephrology, The Tenth People’s Hospital of Tongji University, Shanghai, 200072 China; 5 Department of Epidemiology, School of Public Health of Fudan University, Shanghai, 200032 China; 6 Department of General Practice, Xujiahui Community Healthcare Center of Xuhui District of Shanghai, West Guangyuan Road No.349, Shanghai, 200030 China

**Keywords:** Cancer genetics, Oncogenes, Tumour biomarkers

## Abstract

Kidney renal clear cell carcinoma (KIRC) is the most prevalent type of kidney cancer and causes thousands of deaths each year. The prognosis for KIRC is poor. One critical factor is that the mechanism beneath KIRC is unclear. ORM1 is a reactant to acute inflammation. In this study, we demonstrated that methylation of ORM1 promoter was low and ORM1 was expressed significantly higher in KIRC. KIRC with higher ORM1 expression exhibited worse survival probability. Meanwhile, ORM1 was expressed higher in KIRC cell lines. When ORM1 was knocked down, cell proliferation ability was inhibited potently compared to the NC control. Cell migration as well as invasion ability were also suppressed dramatically. At molecular level, the expression of active caspase-3 and Bax was upregulated in ORM1-KD group while Bcl-2 downregulated. Moreover, CALR decreased following ORM1-KD and rescued expression of CALR increased Bcl-2 level but reduced the level of cleaved caspase-3 and Bax. Consistently, the apoptotic rate of 786-O and Caki-2 cells was upregulated in ORM1-KD but downregulated after CALR overexpression. The activity of caspase-3 was also regulated by ORM1-KD. In addition, the inhibition rate of sorafenib was enhanced in ORM1 KD group but reduced after overexpression of ORM1. Conclusively, ORM1 is clinically associated with progression of KIRC and regulates cell proliferation, migration, invasion, and apoptosis in KIRC. Moreover, ORM1 affects the efficiency of sorafenib in KIRC and regulates caspase-3 mediated cascades response through CALR molecule. This study provides us a new way to recognize the development and progression in KIRC.

## Introduction

Kidney cancer is one of the top six malignant urogenital cancers for men worldwide, which accounts for about 5% of all cancers in males^1^. Renal cell carcinoma originating from renal tubular epithelial cells accounts for over 90% of kidney cancers. And kidney clear cell carcinoma (KIRC) is the most common type in renal cell carcinoma^2^. Epidemically, several thousands of people die of KIRC each year. However, surgical removing is still the major therapy for KIRC in clinics while adjuvant and targeted therapy for a small cohorts of patients. The risk factors for KIRC include age, gender, obesity, genetics, and so on^2, 3^. Next-generation sequencing technology demonstrates that hundreds of genetic alterations exist in genome from KIRC patients, which suggests the pivotal roles of genetics in tumor progression.

As is known, tumor microenvironment in solid cancers is characterized by immunosuppressive treats and this assists the evading of tumor cells from killing by immune cells. Orosomucoid 1 (ORM1) is an acute-phase reactant to acute inflammation and exhibits immunosuppressive roles, which might be a risk factor of tumor^4^. Indeed, ORM1 has been reported to be associated with prognosis or progression of tumor. For example, higher expression of ORM1 predicted worse overall survival in colorectal cancer patients^5^. In hepatocellular carcinoma (HCC), ORM1 was highly correlated with tumor grade and vascular invasion^6^. Qiong et al.^7^ reported that ORM1 contributed to progression of breast cancer by regulating production of inflammatory factors. ORM1 was also shown to be a biomarker or predictor in prostate cancer, bladder cancer, and lung cancer^8–10^. In addition, ORM1 was shown to promote tumor angiogenesis and metastasis^11, 12^. And ORM1 contributed to chemotherapeutic drug resistance in HCC and breast cancer^6, 13^. In a word, ORM1 is involved in the progression, metastasis, and drug resistance in cancers. However, the role of ORM1 in KIRC is unknown at present. Moreover, the internal mechanism of ORM1 underpinning KIRC is still a puzzle.

To elucidate the role and the mechanism of ORM1 in KIRC, we analyzed the clinical importance of ORM1 in KIRC based on TCGA database. Then ORM1 was knocked down in KIRC cell lines, and cell behaviors were determined subsequently. After that, the downstream responsive signaling pathway was explored.

## Materials and methods

### TCGA data analysis

The protein expression and promoter methylation level of ORM1 in KIRC was analyzed from the TCGA database (http://ualcan.path.uab.edu/analysis.html). The survival probability of KIRC patients with high ORM1 expression (n = 133) and low/medium expression (n = 398) was analyzed based on the TCGA database.

### Cell lines and cell culture

KIRC cell lines including 786-O, A498, and Caki-2 cells were obtained from ICell Bioscience Inc, Shanghai (Shanghai, China). 786-O and A498 cell lines were cultured in DMEM medium (KeyGen Biotech, Nanjing, China) containing 10% FBS (Gibco, USA). Caki-2 cells were cultured in RPMI1640 medium (KeyGen Biotech, Nanjing, China) containing 10% FBS. All cells were kept in atmosphere with 5% CO_2_ at 37℃.

### Synthesis and transferring of small interfering RNA and recombinant plasmid carrying ORM1 or CALR

Small interfering RNA targeting ORM1 gene (ORM1-KD: sense: 5′-UUAUUGUACUCCUCGUUUCGA-3′, antisense: 5′-GAAACGAGGAGUACAAUAAGU-3′) or negative control (NC: sense: 5′-UUACUCCUCGAUUGUACGACGA-3′, antisense: 5′-GUCGUACAAUCGAGGAGUAAGU-3′) was synthesized by General Biosystem (Anhui, China). Then the ORM1-KD was transferred into RCC cell lines with lipo2000 reagent (Ranyan Bio, Shanghai, China) according to the instructions. In brief, 50 pmol of ORM1-KD or negative control (NC) was mixed with 5 μl of lipo2000 reagent for 20 min at room temperature. Cells were pretreated with culture medium with 2% FBS for 2 h. Then the mixture was added into the cells and cultured for 6 h. Fresh medium was added into the cells and cultured for another 48 h. Cells were collected for following experiments.

The coding sequences for ORM1 and CALR were synthesized by General Biosystem, subcloned into vector pcDNA3.1, and confirmed by sequencing, respectively. Then 2 μg of the recombinant plasmids were transferred into RCC cell lines as stated above.

### Cell counting kit-8 assay

KIRC cell lines were plated into 96-well plates and treated with siORM1 as the above. CCK-8 reagent was added into each well at 24, 48, 72, 96 h and cells were kept for another 1 h. Then the absorbance value at 450 nm wavelength was detected on a microplate reader.

### Transwell assay

To investigate cell migration ability, KIRC cell lines were treated with siORM1 for 6 h, seeded into the transwell insert with 8 μm pore, and placed into 24-well plates with culture medium containing 10% FBS. Culture medium with no FBS was added into the up inserts. After 24 h, the cells on the upper surface of the insert were scraped gently and the inserts were fixed in 4% formaldehyde for 10 min at room temperature. Then the inserts were stained in 0.05% crystal dye for 10 min at room temperature. After washing with PBS for three times, the inserts were dried, photographed, and the relative cell migrating rate was analyzed.

To investigate cell invasion ability, transwell insert was pretreated with 50 μl Matrigel (BD Bioscience, USA) for 24 h at 4 ℃. Then cells were treated as the above.

### Detection of cell apoptosis by FACS method

KIRC cell lines were seeded into 6-well plates and treated with siORM1 as the above. After 48 h, cells were collected and washed with cold PBS. Then cells were stained with 100 μl binding buffer containing 5 μl ANNEXIN V/FITC and 10 μl PI dye for 15 min at room temperature. 400 μl binding buffer was added before detected by flow cytometry method (Beckman Coulter, USA).

### Detection of caspase-3 activity

RCC cell lines were seeded into 24-well plates at 50,000/well and treated as the above. After 48 h, cells treated according to the instructions of Caspase-3 colorimetric Assay Kit (NobleRyder, Beijing, China). Briefly, cells were collected and lysed with lysis buffer, ice-cold for 10 min, and centrifugated at 1000 g for 5 min. Then 10 μl of the supernatant was collected and transferred into 96-well plates. After substrates were added into the 96-well plates and placed at 37℃ for 2 h, the absorbance value at 405 nm wavelength was detected on the microplate reader and the activity of caspase-3 was calculated as below: OD405_test_/OD405_control_ × 100%.

### Quantitative real-time PCR assay

KIRC cells were collected and washed with cold PBS. Total RNA from KIRC cells were extracted with RNA easy Kit (Beyotime, China) under manufacturer’s instructions and quantified on Onedrop equipment (Hangzhou, China). 0.5 to 1.0 μg total RNA was used as template for synthesize of first strain of cDNA. Then 1 μl product was used to quantify the expression of candidate genes. β–actin was used as internal control. The primer sequences of CALR and ORM1 were as below: CALR: forward primer: 5′-GGCAGATCGACAACCCAGAT-3′, reverse primer: 5′-CACGTCTCGTTGCCAAACTC-3′; ORM1: forward primer: 5′-ACCTACATGCTTGCTTTTGACG-3′, reverse primer: 5′- CCCCCAAGTCTCTGTCCTGA-3′. The protocols are as below: 95℃, 5 min; (95℃, 15 s; 60 °C, 20 s) for 40 cycles.

### Western blotting assay

KIRC cells were collected and washed with cold PBS. Total proteins were extracted from Protein Extracting Kit (Beyotime, China) under manufacturer’s instructions and quantified on Onedrop equipment (Hangzhou, China). Then 10 μg protein was analyzed by SDS PAGE electroporasis and transferred onto cropped PVDF membranes (Millipore, USA) according to the molecular weight of target proteins. The PVDF membranes were blocked with 5% non-fat milk for 1 h at room temperature, cocultured with primary antibodies targeting candidate proteins for 12 h at 4 °C. Then the membranes were washed with PBS and cultured with secondary anbibodies tagged with HRP for 1 h at room temperature. ECL kit was used to detect the protein bands on Tanon 4800 (Shanghai, China). The primary antibodies were purchased from the Abcam, and the informations were as following: ORM1 (ab134160, 1:5000), CALR (ab92516, 1:2500), Bcl-2 (ab32124, 1:1000), Bax (ab32503, 1:5000), cleaved caspase-3 (AC033-1, 1:1000), GAPDH (ab9485, 1:1000), HRP-conjugated goat anti-rabbit IgG (A0208, 1:10,000, Beyotime, China).

### Statistical analysis

All data in this study were shown as mean ± standard derivation based on three repeats. SPSS 16.0 software was used to analyze the significance of each data. Student’s t test was used to compare the difference between two groups. And One-way ANOVA with Tukey’s post hoc test was used to analyze the differences among over three groups. *P* < 0.05 suggests statistically significant difference.

## Results

### ORM1 is clinically correlated with KIRC

**Figure 1:**
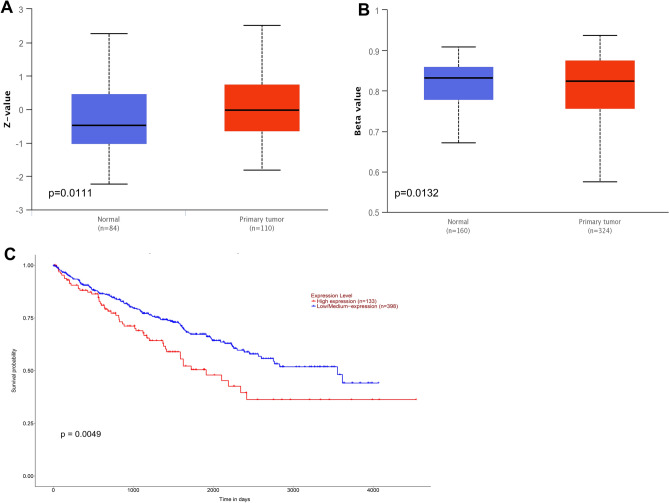
ORM1 was clinically associated with KIRC. (**A**) The expression of ORM1 protein was much higher in primary tumor tissues than normal tissues. (**B**) The methylation level of ORM1 promoter was lower in primary tumor tissues than normal tissues. (**C**) The survival probability of KIRC patients with high ORM1 expression was lower than those with low ORM1 expression.

Based on the data from the TCGA database, we displayed that the protein expression level of ORM1 in primary tumor tissues (n = 110) was significantly stronger than the normal tissues (n = 84) (Fig. 1a). In contrast, the methylation level of ORM1 promoter was much lower in primary tumor tissues (n = 324) than the normal tissues (n = 160) (Fig. 1b). Furthermore, the survival probability of patients with high ORM1 expression (n = 133) was significantly worse than patients with low ORM1 expression (n = 398) (Fig. 1c). Therefore, ORM1 showed clinical implication in KIRC patients.

### ORM1 is essential to cell proliferation in KIRC

**Figure 2:**
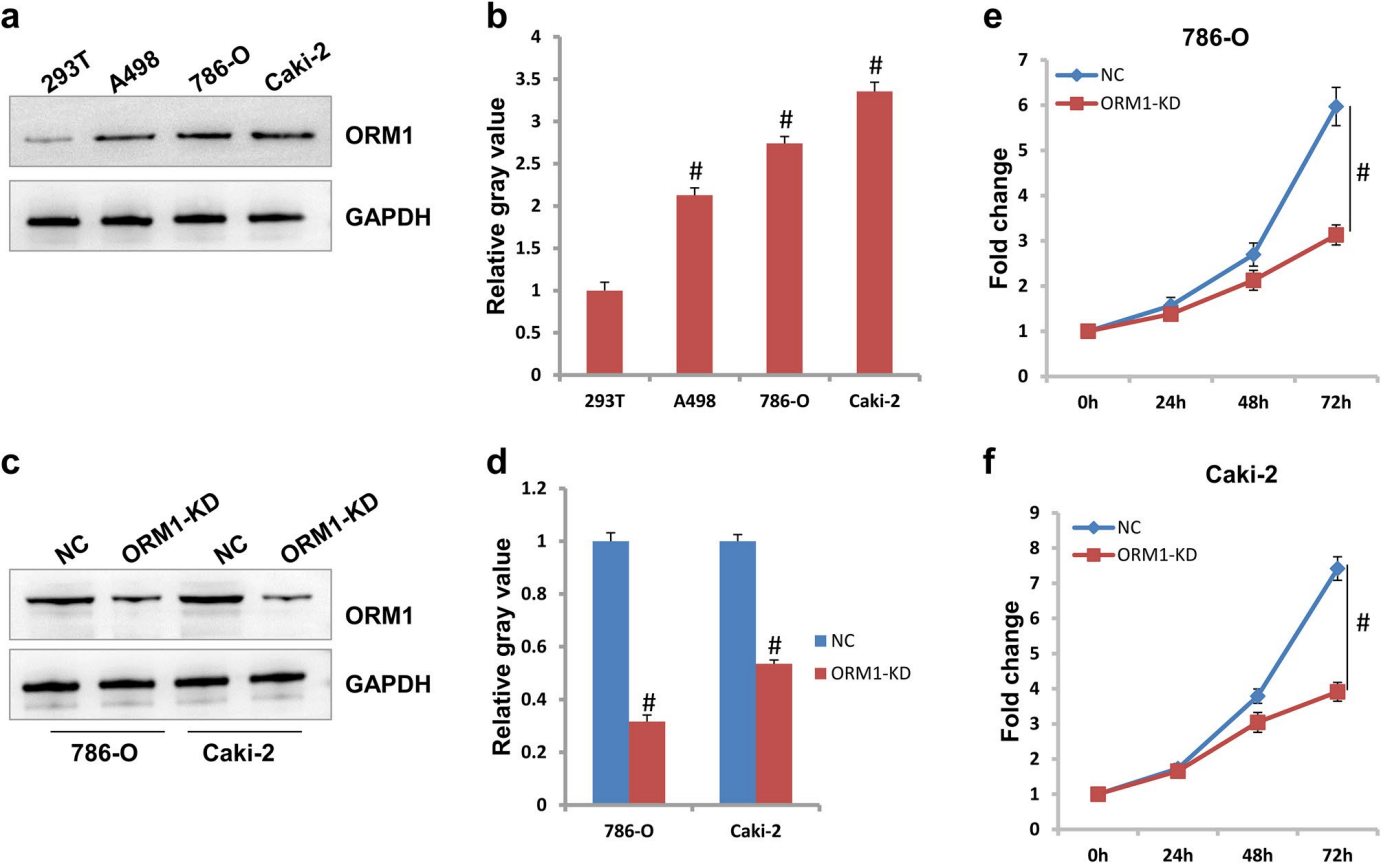
ORM1 was essential to cell proliferation. (**a**) The expression of ORM1 protein in A498, 786-O, and Caki-2 cells was much higher than the 293 T cells. (**b**) The gray value analysis of protein in (**a**). (**c**) ORM1 was knocked down in 786-O and Caki-2 cells. (**d**) The gray value analysis of protein in (**c**). (**e**) Cell proliferation of 786-O cells was inhibited in ORM1-KD group compared to NC group. (**f**) Cell proliferation of Caki-2 cells was inhibited in ORM1-KD group compared to NC group. #*p* < 0.05 showed statistically difference.

As shown in Fig. 2a,b, the protein expression of ORM1 in cell lines including 786-O, A498, and Caki-2 cells were much higher than in the control cells 293 T. To investigate the role of ORM1 in KIRC, ORM1 was knocked down by siRNA targeting to ORM1. As shown in Fig. 2c,d, the expression level of ORM1 in 786-O and Caki-2 cells decreased approximately 36% and 66% compared to NC control, respectively. Unsurprisingly, cell growth ability was destroyed dramatically following ORM1 knockdown. At 72 h, cell proliferation in 786-O cells with ORM1-KD was approximately 53.7% of that in NC control while it was approximately 51.9% in Caki-2 cells (Fig. 2e,f).

### ORM1 deficiency suppresses cell migration and cell invasion

**Figure 3:**
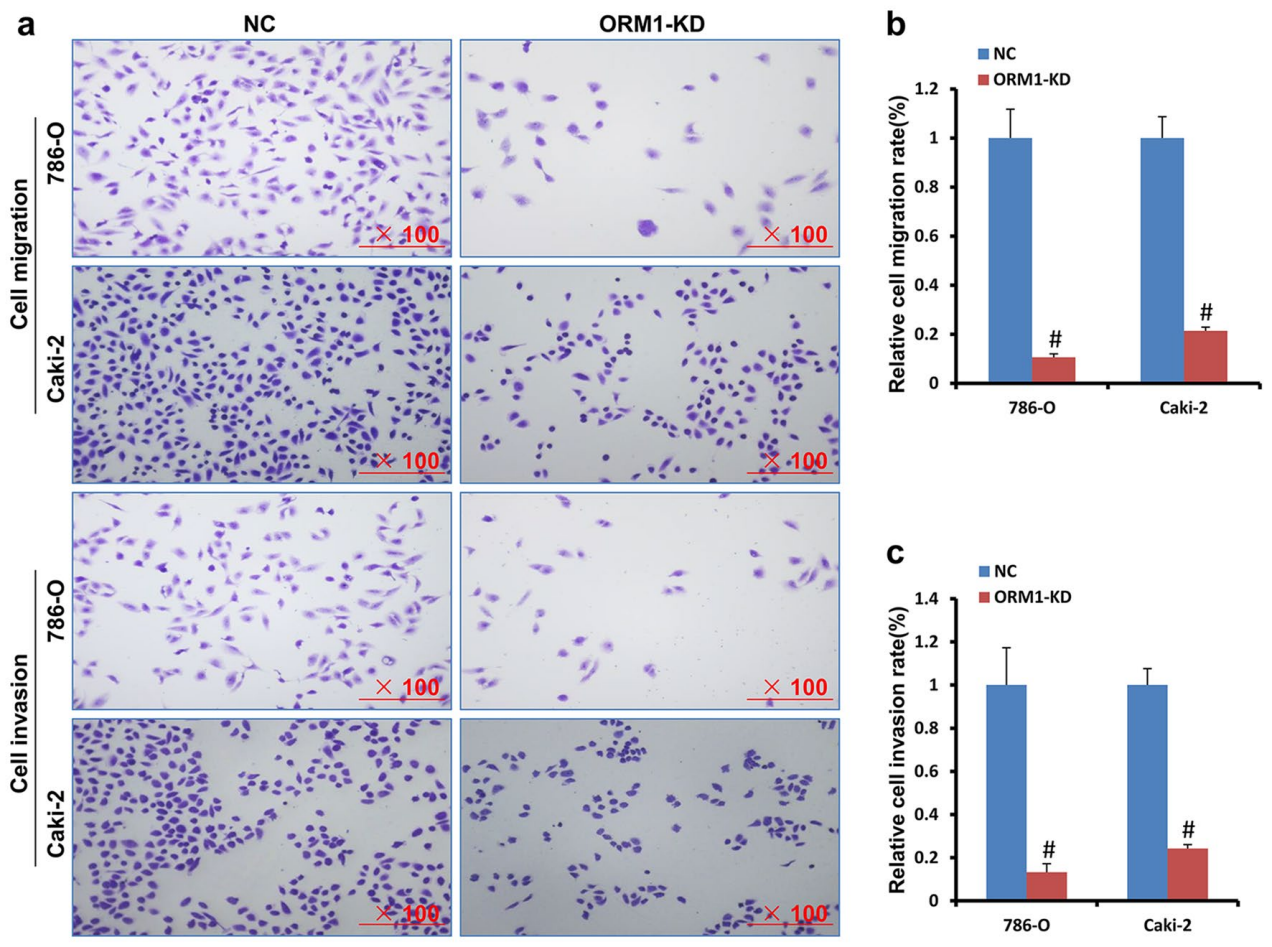
ORM1 was essential to cell migration and invasion. (**a**) Cell migration and invasion was suppressed in ORM1-KD group compared to NC group in 786-O and Caki-2 cells in transwell assay with/without Matrigel. (**b**) The statistical analysis of cells in cell migration in (**a**). (**c**) The statistical analysis of cells in cell invasion in a. #*p* < 0.05 showed statistically difference.

Metastasis is one of the typical characteristics in cancer including KIRC. In this study, we demonstrated that cell migration ability in 786-O cell with ORM1-KD exhibited approximately 89.4% reduction compared to the NC control (Fig. 3a,b). In Caki-2 cells, cell migration ability reduced by about 78.6% in KD group compared to NC control (Fig. 3a,b). In consistent, cell invasion ability was reduced by about 86.8% and 75.8% in 786-O and Caki-2 cells compared to NC control, respectively (Fig. 3a,c).

### ORM1 deficiency causes activation of caspase-3 cascades pathway and CALR is a mediator

**Figure 4:**
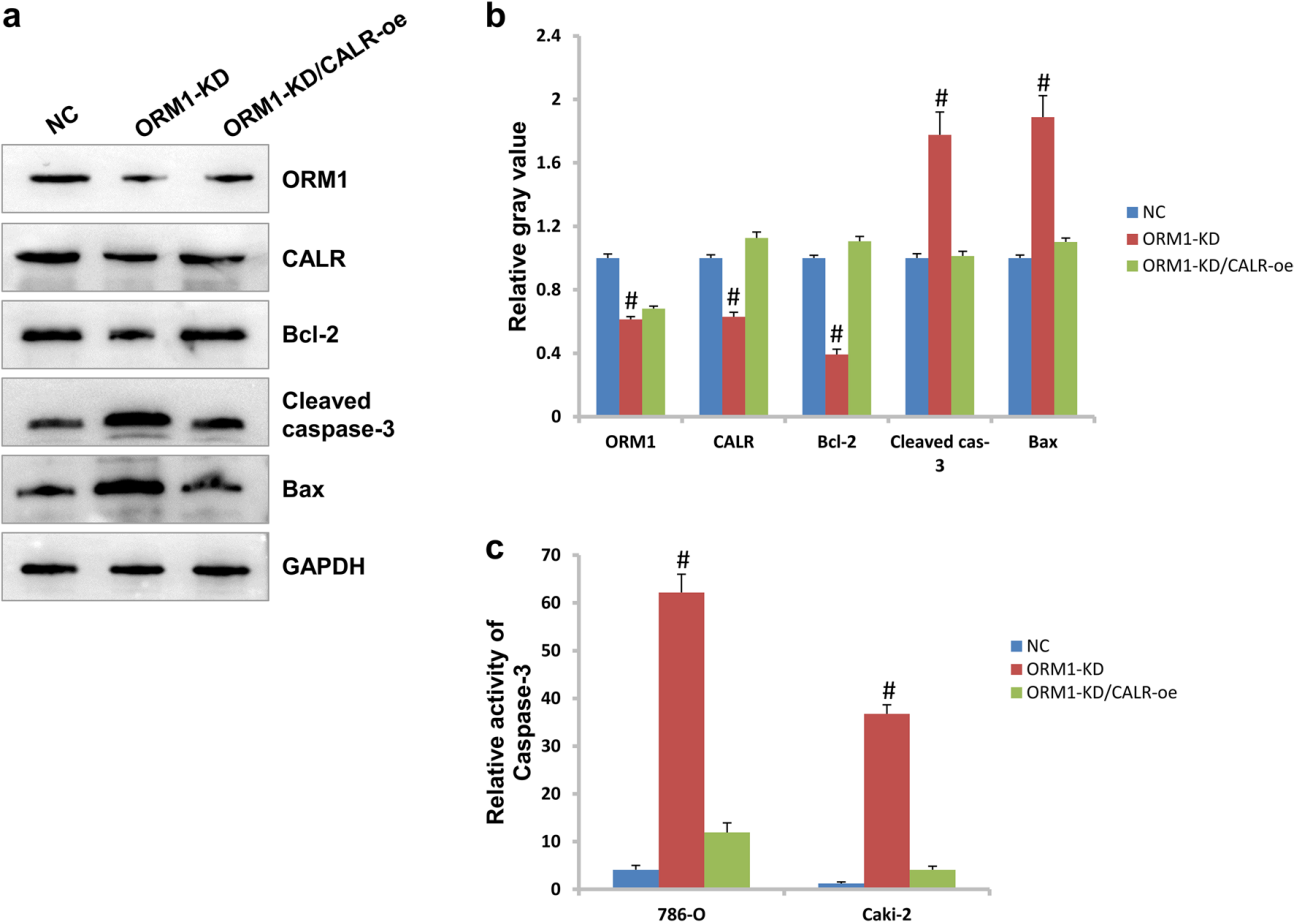
Caspase-3 mediated cascades response was regulated by ORM1. (**a**) The expression of critical members in caspase-3 cascades response was detected in western blotting assay. (**b**) The gray value of proteins in (**a**). (**c**) The activity of caspase-3 was detected by ELISA method. #*p* < 0.05 showed statistically difference.

To explore the internal mechanism of ORM1 in KIRC, we detected the common signaling molecules involving in cell growth, survival, and metastasis. Unexpectedly, we found that caspase-3 mediated cascades responses were the downstream responding signaling pathway. As shown in Fig. 4a,b, the protein level of cleaved caspase-3 exhibited 1.8 fold raise in ORM1-KD group compared to NC group. The expression level of Bax was upregulated by approximately 1.9 fold. In contrast, Bcl-2 expression was reduced by approximately 65% in ORM1-KD cells compared to NC control. Moreover, we found that the expression of CALR was reduced by about 51% in ORM1-KD cells. CALR was a molecule interacting with ORM1 based on String database. When CALR was overexpressed in ORM1-KD cells (Fig. 4a,b and Fig. S1a), the expression of cleaved caspase-3 and Bax was reduced but Bcl-2 increased, which suggested the activity of caspase-3 was inhibited.

As shown in Fig. 4c, the activity of caspase-3 was upregulated by about 15 fold when ORM1 was knocked down in 786-O cells but downregulated dramatically after overexpression of CALR (CALR-oe).

**Figure 5:**
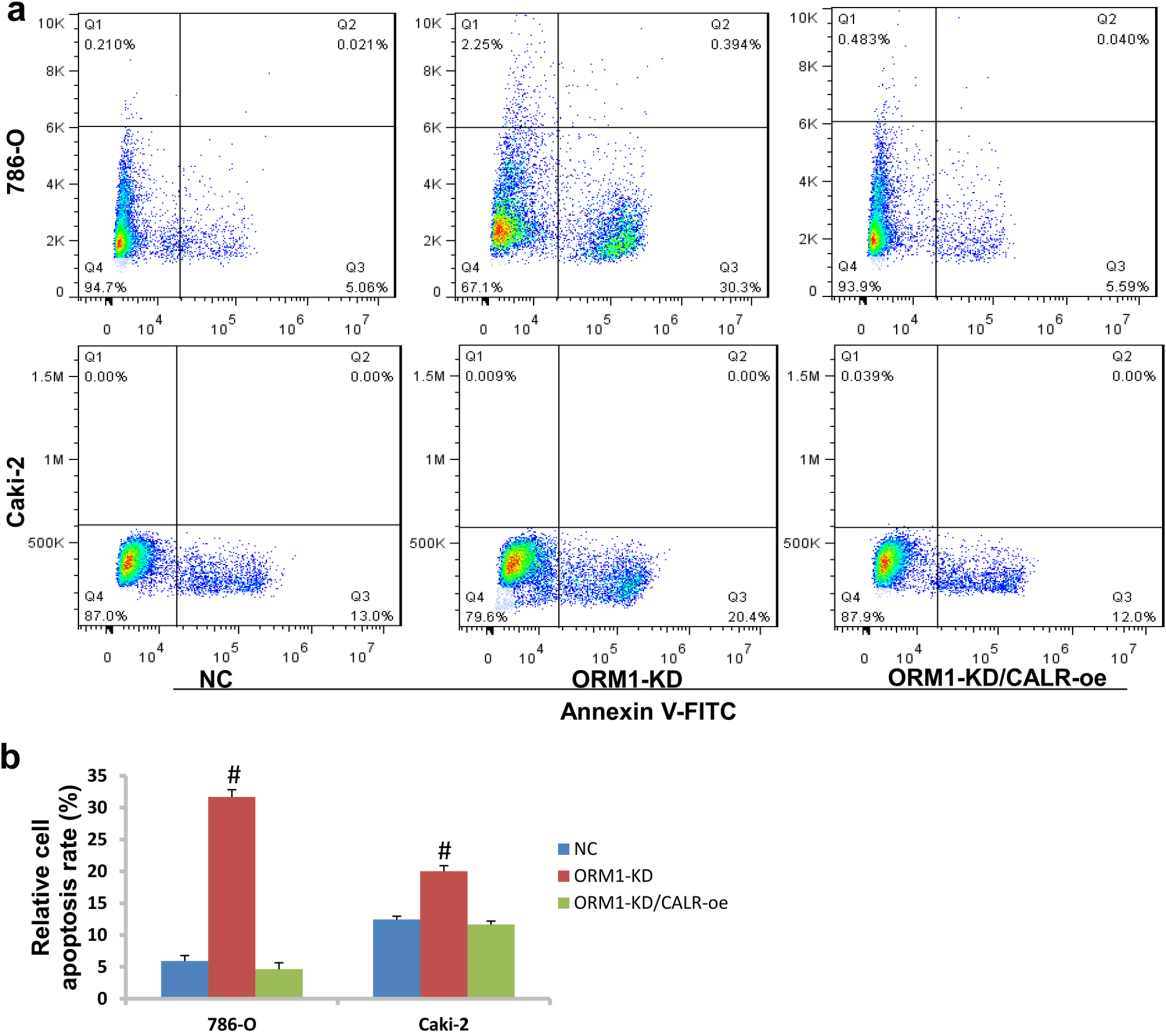
ORM1 regulated cell apoptosis through CALR in KIRC. (**a**) Cell apoptosis was detected by Annexin V/FITC dye method. (**b**) The statistical analysis of cells in cell migration in (**a**). #*p* < 0.05 showed statistically difference.

In consistent, cell apoptosis rate in 786-O cells was 31.65% after ORM1 knockdown while it was 20% in Caki-2 cells, which was significantly higher than NC control (Fig. 5a,b). But overexpression of CALR significantly reduced cell apoptotic rate of cells with ORM1-KD.

### ORM1 knockdown resensitize KIRC cells to Sorafenib

**Figure 6:**
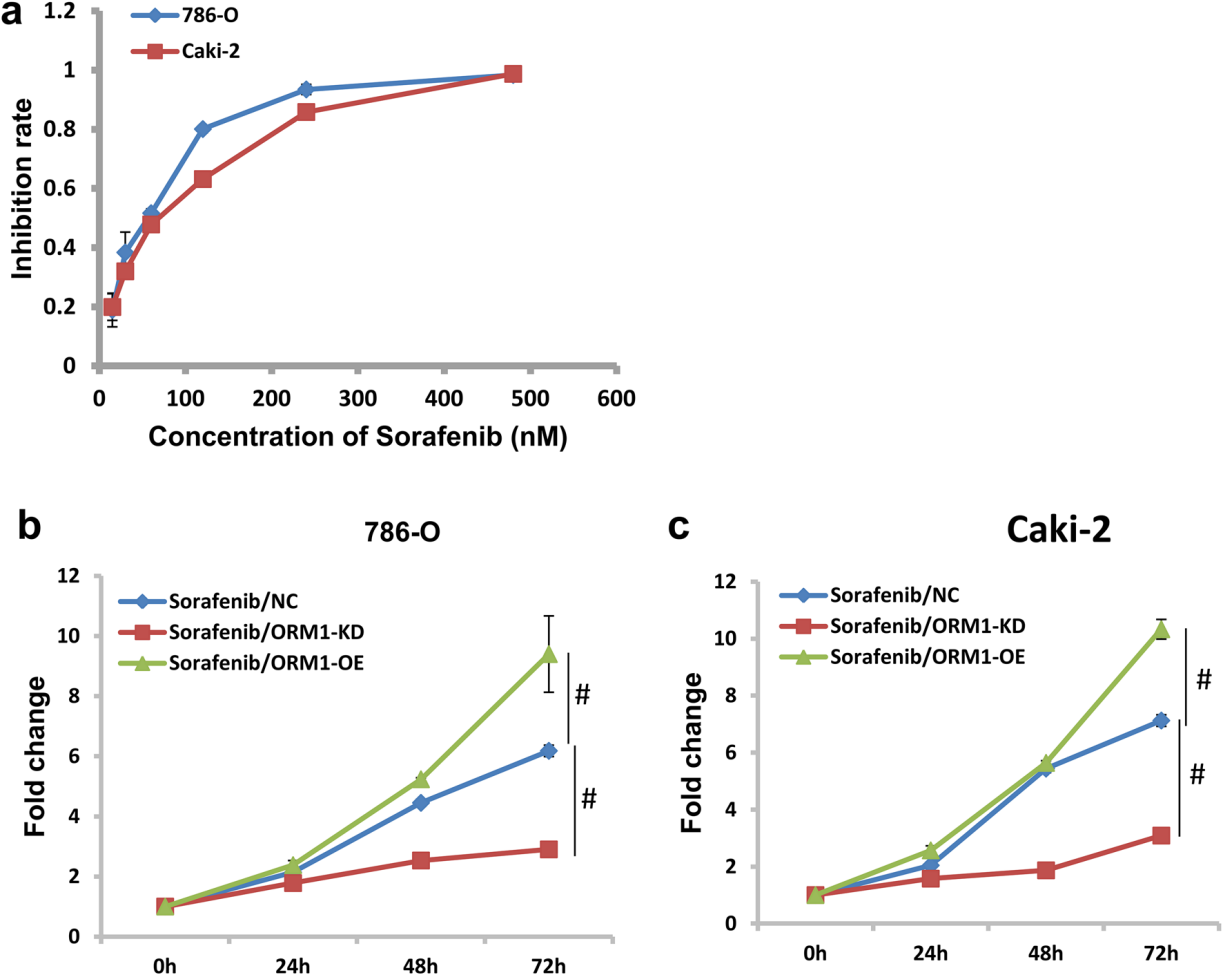
ORM1 enhanced the efficiency of sorafenib in KIRC. (**a**) Sorafenib inhibited cell proliferation in concentration-dependent manner. (**b**) The efficiency of sorafenib was enhanced in ORM1-KD group but relieved in ORM1-OE group in 786-O cells. (**c**) The efficiency of sorafenib was enhanced in ORM1-KD group but relieved in ORM1-OE group in Caki-2 cells. #*p* < 0.05 showed statistically difference.

Sorafenib is a clinical drug for target therapy of renal cell carcinoma. As shown in Fig. 6a, sorafenib exhibited potent inhibitory effects on cell growth in KIRC. The IC50 value of sorafenib in 786-O cells was 48.8 nM but 60.6 nM in Caki-2 cells (Fig. 6a). When ORM1 was downregulated, sorafenib exhibited more potent inhibition on cell growth in 786-O and Caki-2 cells. As shown in Fig. 6b,c, the inhibition rate of sorafenib was upregulated by approximately 41.5% and 63.4% in 786-O and Caki-2 cells, respectively. In contrast, the inhibitory role of sorafenib was significantly released after ORM1 was overexpressed (Fig. 6b,c and Fig. S1b).

## Discussion

Kidney renal clear cell carcinoma is the substantial type of kidney cancer and causes thousands of deaths each year. One of the most critical factors is the lack of recognition of the internal mechanism causing KIRC. Genetic alterations including gene mutation, truncation, SNP, abnormal epigenetic modification have been proved to be vital risk factors in cancer^14^. For example, increased expression of oncogene CEP, mutation of tumor suppressor p53, fusion of BCR-ABL, and decreased DNA methylated, and so on^15, 16^. The importance of genetic alterations is firmly supported by the data from TCGA database. TCGA database is a public resource consists of DNA/RNA sequencing data, clinicopathological factors of nearly all types of cancers^17^. Based on TCGA data, hundreds of aberrations exist in the genome of each cancer but only a single or several types of aberrations which called “Driver” play the dormant roles^17^. In the above text, ORM1 was shown to be expressed abundantly in KIRC tissues and the methylated level of promoter was downregulated compared to the normal tissues based on the TCGA data. DNA methylation is a very important mechanism of gene regulation^16^. In general, declined methylation in promoter CpG islands often activate gene transcription and leads to increased gene expression. So, low methylation level of ORM1 is in consistent with its elevated expression in KIRC tissue. Moreover, KIRC patients with high expression of ORM1 exhibited worse survival ability. Survival time is a critical indicator in cancer and “Driver” gene is often negatively correlated with patient’s survival while tumor suppressor delays the progression of cancer. Therefore, this data suggests that ORM1 was clinically correlated with the formation or progression of KIRC and might be an important prognostic predictor of KIRC.

It is reported that cancer is characterized by ten hallmarks including distant metastasis, unlimited growth, drug-resistance, immune tolerance, and so on^18^. In physiological state, cells undergo growth/senescence/death cycle, and this maintains homeostasis. However, cancer cells could grow and expand without the limit of contact-inhibition if only the energy and space was enough^19^. In clinics, heavy tumor burden exhibits lots of adverse effects on function of normal organs, which brings great threaten to patients. ORM1 was reported to promote cell proliferation and tumor growth in breast cancer and HCC^6, 7^. Consistently, ORM1 was shown to be vital to cell proliferation in KIRC. Furthermore, ORM1 contributed to cell migration as well as invasion in KIRC, which suggests that ORM1 might be involved in the process of tumor metastasis. In cancer, cells invade the surrounding tissues, invade through the blood vessels, and migrate to distant tissues. Indeed, ORM1 was shown to increase migration or vascular invasion in breast cancer and HCC^6, 7^.

Programmed cell death (apoptosis) is an important mechanism of homeostasis maintaining^20^. Generally speaking, cells infected by foreign pathogens or with genetic aberrations need repairing or removing out. Apoptosis is one of the critical ways to remove abnormal tissues. However, tumor cells could avoid being locked by apoptosis and survive with genetic errors. In clinics, chemical drugs were applied to kill tumor cells and activation of cell apoptosis response is the major mechanism^21^. In this study, we demonstrated that the activity of caspase-3 was induced after ORM1 knockdown. At the same time, the expression of Bax was upregulated while Bcl-2 downregulated. Bax was reported to be induced when caspase cascades response was activated^20^. Bcl-2 is a negative regulator of apoptosis and its level declined when apoptosis process is activated^20^. Therefore, ORM1 knockdown leaded to activation of caspase-3-mediated cascades responses in KIRC. And that the apoptotic rate of KIRC cells increased following ORM1 knockdown further supports the activation of apoptosis responses. Meanwhile, we found that CALR was a potential molecule interacting with ORM1 based on String database which is a public database of protein–protein interaction^22^. And forced expression of CALR recovered partially the ability of proliferation and invasion when reduced cell apoptotic rate in ORM1-KD KIRC cells. Furthermore, caspase cascades response was suppressed after CALR overexpression. CALR was an endoplasmic reticulum protein and was responsible for the maintenance of cell proteostasis. However, CALR mutations were shown to favor oncogenesis by impairing cell homeostasis and compromising immunosurveillance^23^. Moreover, CALR was reported to regulate apoptosis process in cancers. For example, Salati et al.^24^ demonstrated that CALR mutation decreased sensitivity of K562 cells to oxidative stress and inhibited apoptosis. In nasopharyngeal carcinoma, RCN2 curbed mitochondrial apoptosis and interacted with CALR^25^. In this study, after CALR overexpression, the expression levels of cleaved caspase-3 and Bax were reduced but Bcl-2 increased. This means that the caspase-3 mediated apoptosis effect of ORM1 knockdown in KIRC was suppressed by CALR. That the apoptotic rate of KIRC cells was reduced in ORM1-KD cells after CALR was overexpressed further supported this foundation at molecular level. Therefore, ORM1 plays negative role in apoptosis of KIRC and CALR is a critical mediator in this process.

Sorafenib is the first-line clinical drug applied in renal cell carcinoma^26^. In this study, we demonstrated that ORM1 might be a potential target to affect the effect of sorafenib in KIRC. The effect of sorafenib on KIRC cells was enhanced when ORM1 decreased. In contrast, the effect of sorafenib was relieved after ORM1 overexpression. These data suggest that interfering ORM1 could strengthen the killing effects of sorafenib on KIRC cells, which might relieve the adverse effects on normal tissues. Sorafenib was reported to induce cell apoptosis in cancer. However, both ORM1 and CALR were shown to negatively regulate cell apoptosis in KIRC in the above. And CALR was proved to be an important mediator downstream of ORM1. Therefore, it is possible that CALR might be also closely related to the efficacy of Sorafenib in KIRC and artificial knockdown of CALR might increase the effects of Sorafenib in KICR patients. However, it needs much more work to elucidate the relationship of ORM1, CALR, and Sorafenib in KIRC, which will be our emphasis in the following days.

In summary, ORM1 was overexpressed in KIRC and associated with survival time. ORM1 was essential to cell proliferation, migration, invasion and apoptosis in KIRC. ORM1 regulated caspase-3-mediated cascades responses, CALR was a critical downstream pathway from ORM1 to caspase, and knockdown of ORM1 could enhance the inhibitory role of sorafenib in KIRC.

### Supplementary Information

Supplementary Figures.

Supplementary Figure S1.

## Data Availability

The datasets generated during and/or analyzed during the current study are available from the corresponding author on reasonable request.
