# Supplementary material for: ORM1 promotes tumor progression of kidney renal clear cell carcinoma (KIRC) through CALR-mediated apoptosis
Source: Sci Rep. 2023 Sep 21;13:15687. doi: 10.1038/s41598-023-42962-w (PMC10514263; doi:10.1038/s41598-023-42962-w)

Prestained marker(10-180kDa)

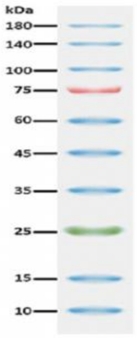

For Figure 2A:

ORM1: 45 kDa

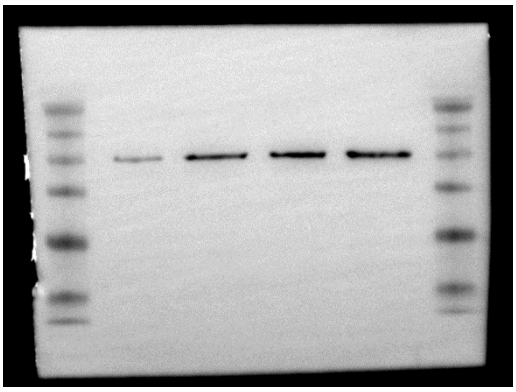

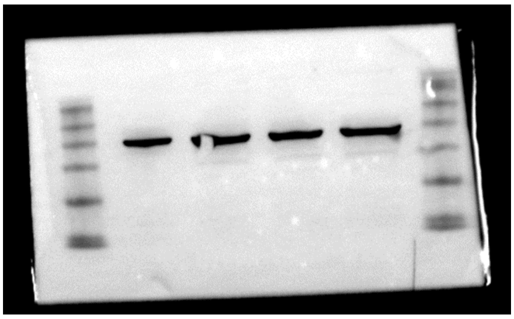

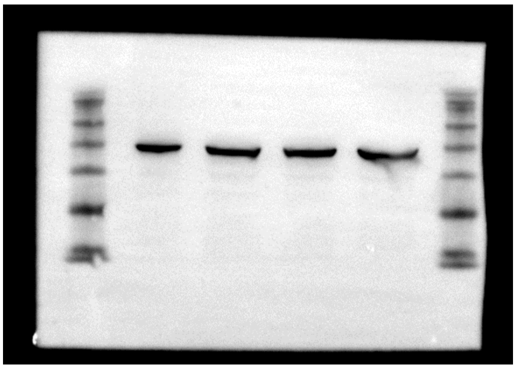

GAPDH: 36 kDa

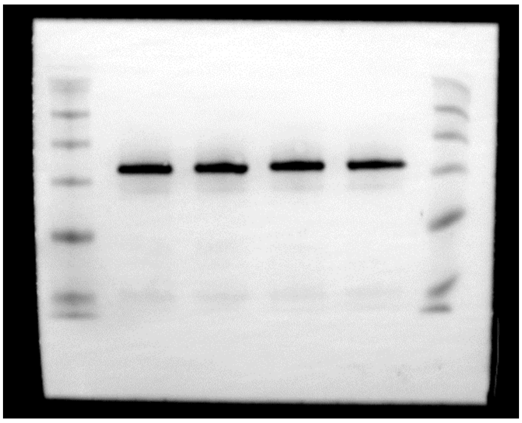

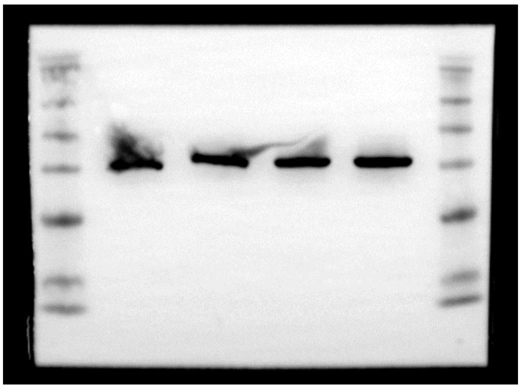

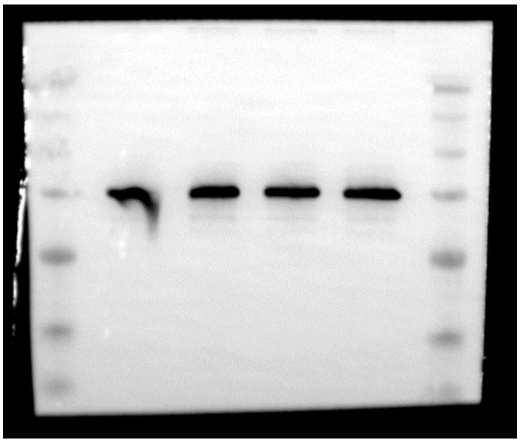

For Figure 2C:

ORM1: 45 kDa

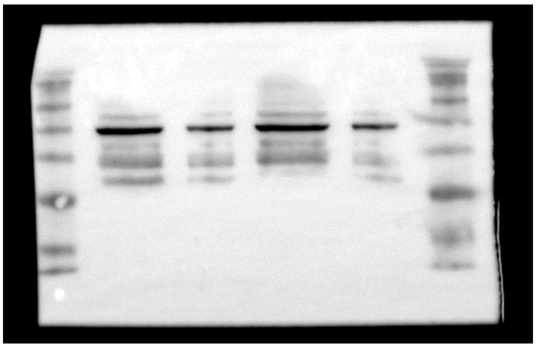

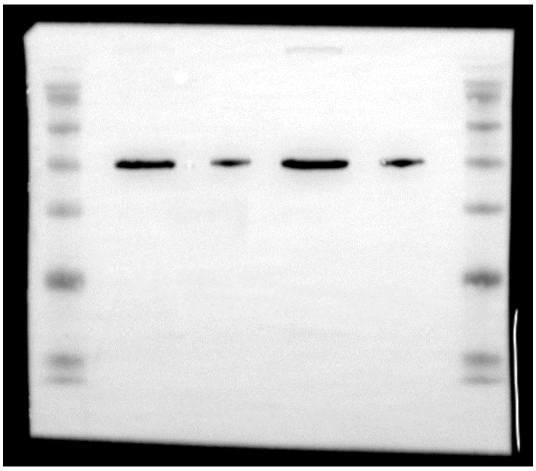

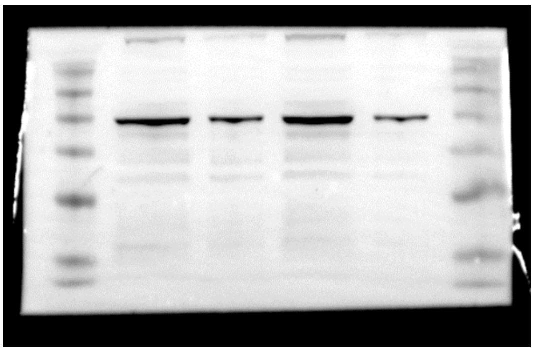

GAPDH: 36 kDa

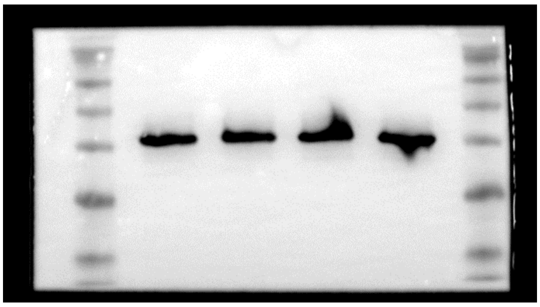

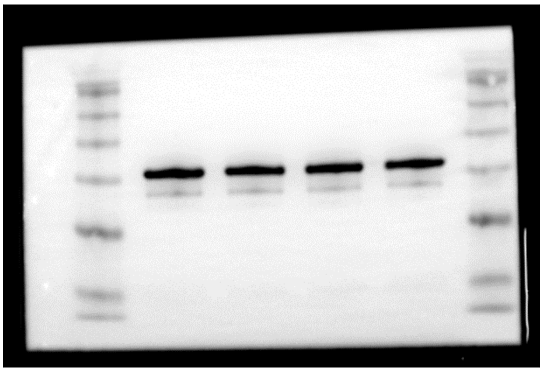

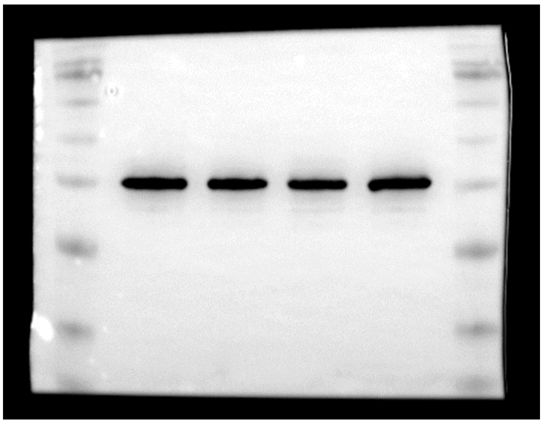

For Figure 4A:

ORM1: 45kDa

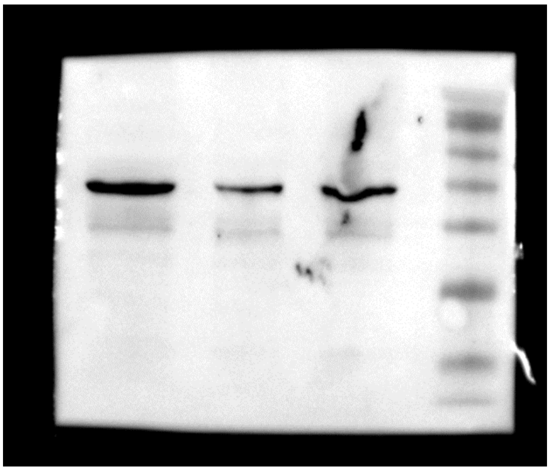

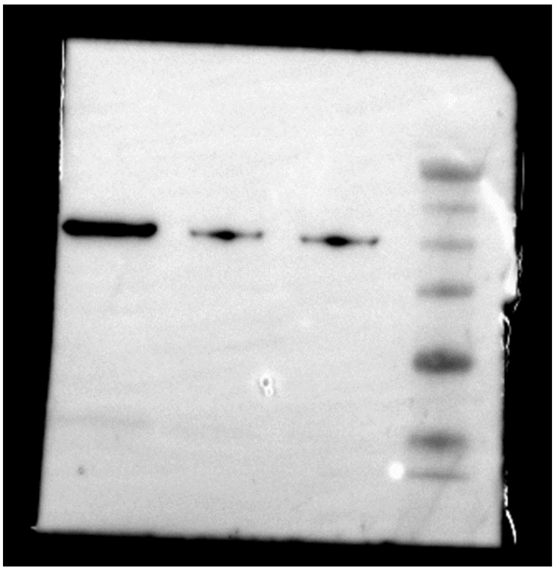

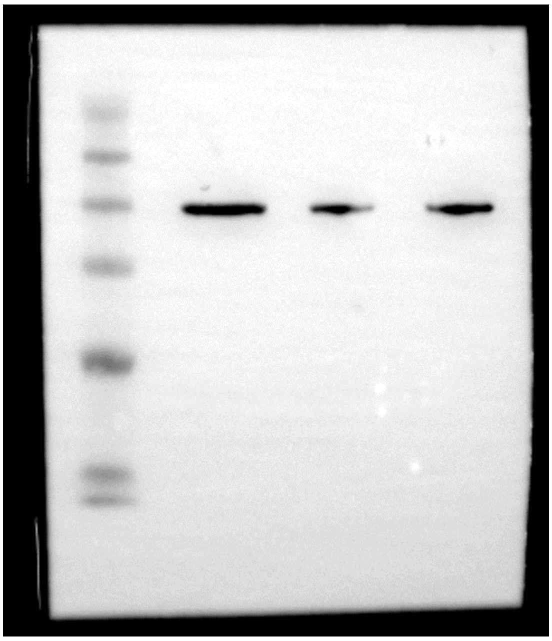

CALR: 48kDa

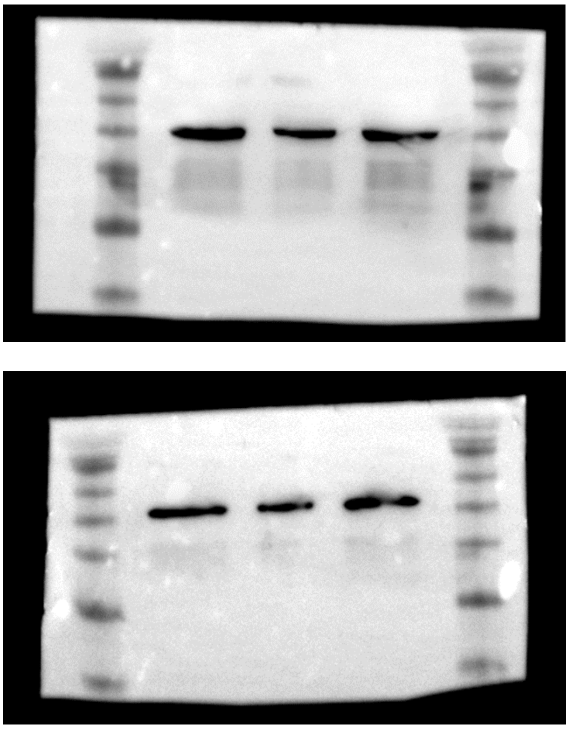

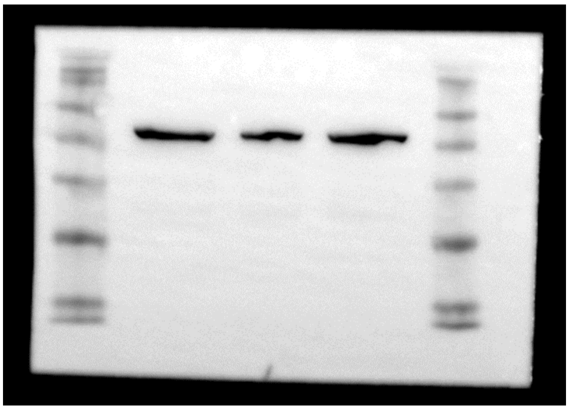

Bcl-2:27kDa

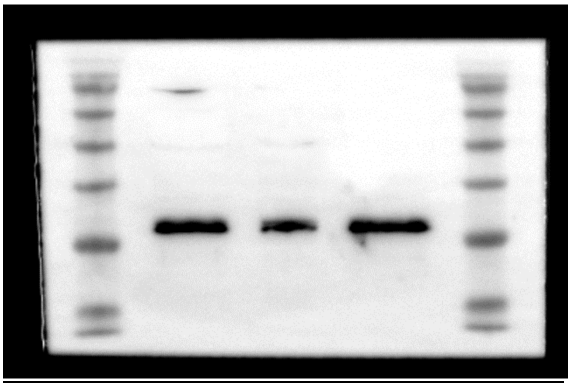

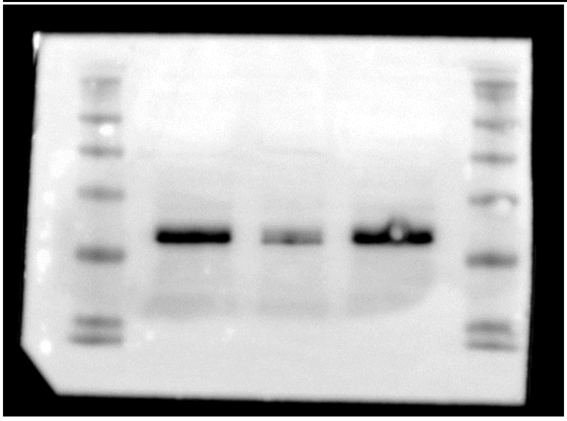

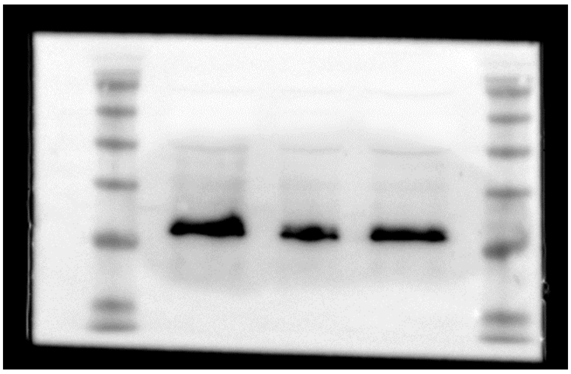

cleaved caspase-3: 17 kDa

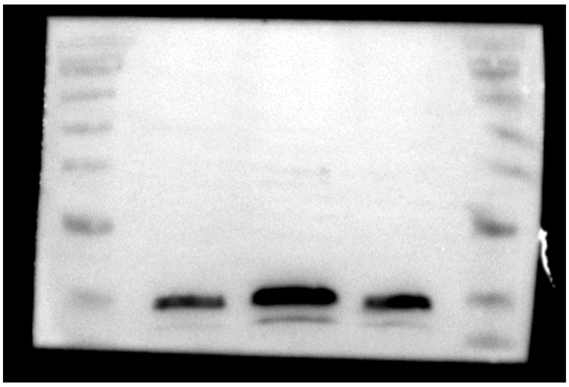

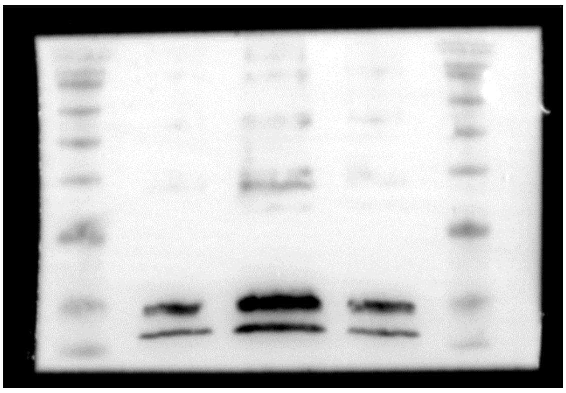

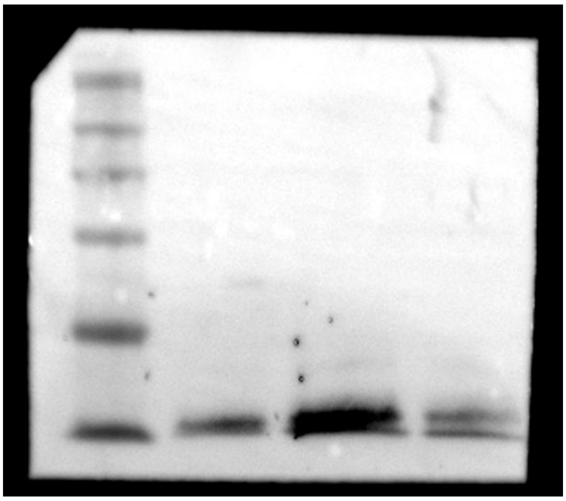

Bax: 21 kDa

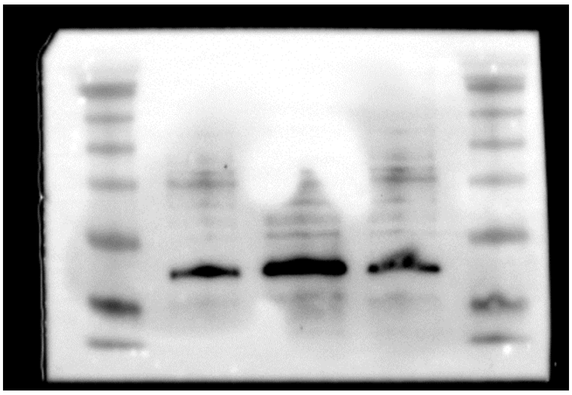

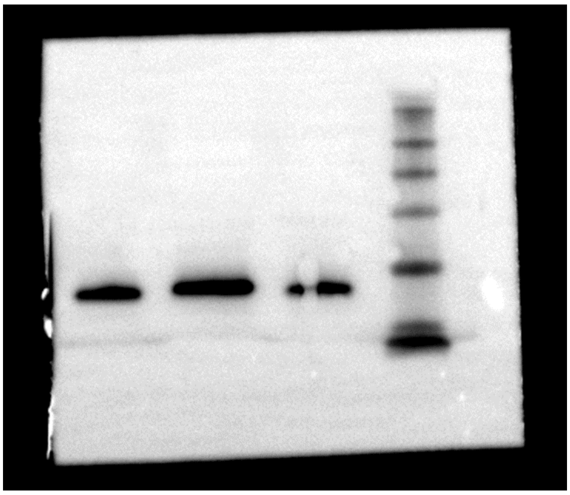

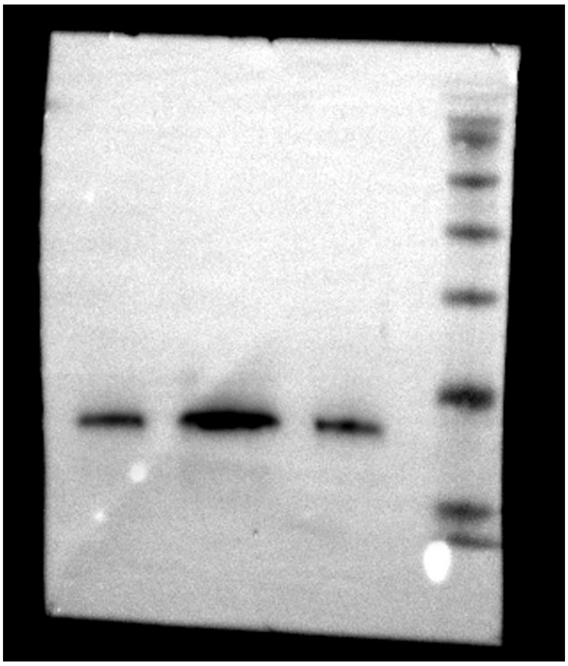

GAPDH:36 kDa

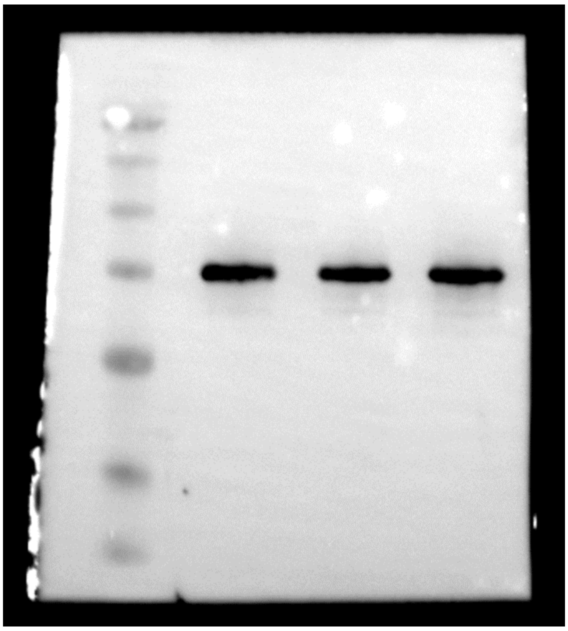

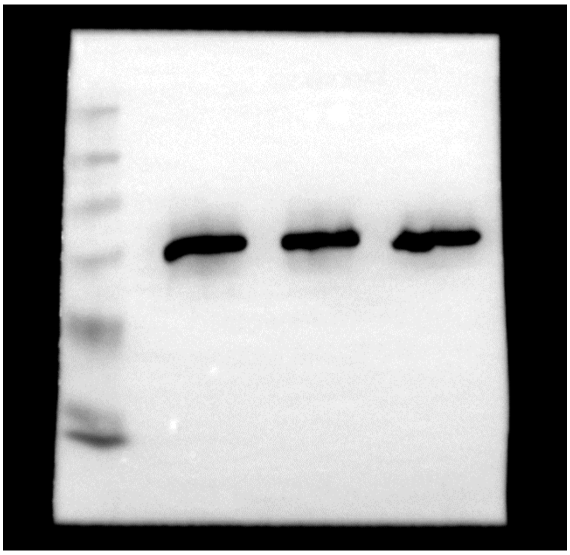

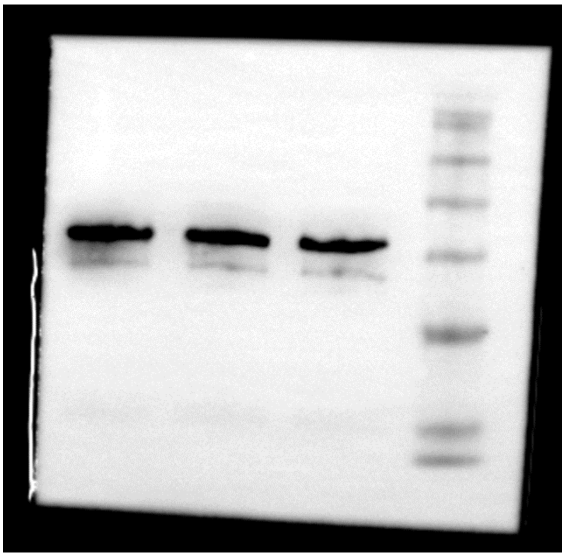

Supplement: Supplementary file 1 — Supplementary Figures. [file 41598_2023_42962_MOESM1_ESM.pdf]
