# Supplementary material for: ORM1 promotes tumor progression of kidney renal clear cell carcinoma (KIRC) through CALR-mediated apoptosis
Source: Sci Rep. 2023 Sep 21;13:15687. doi: 10.1038/s41598-023-42962-w (PMC10514263; doi:10.1038/s41598-023-42962-w)
Supplement: Supplementary file 2 — Supplementary Figure S1. [file 41598_2023_42962_MOESM2_ESM.pdf]

For Figure S1

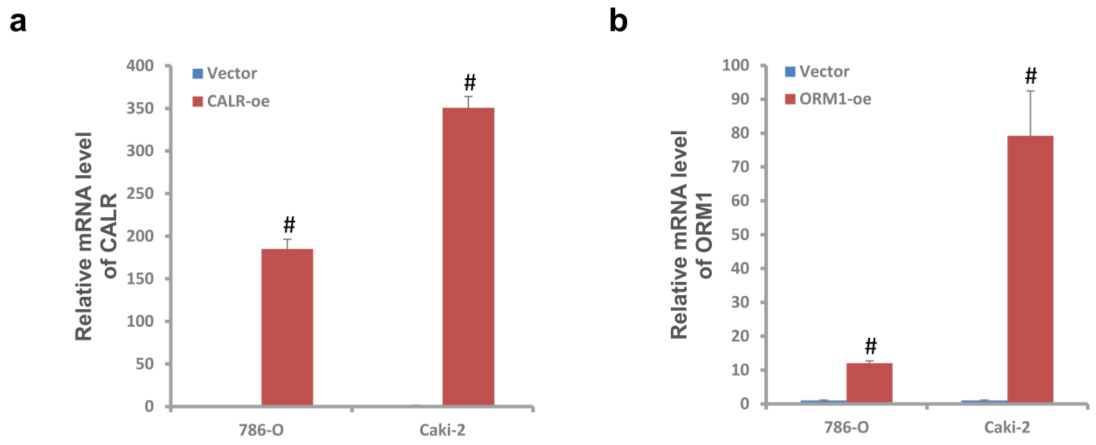

Figure S1. The expression of CALR and ORM1 in RCC. a.CALR was overexpressed in 786-O and Caki-2 cells at mRNA level. b. ORM1 was overexpressed in 786-O and Caki-2 cells at mRNA level. #p ＜0.05 showed statistically difference.
